# Circumcision and its alternatives in Germany: an analysis of nationwide hospital routine data

**DOI:** 10.1186/s12894-021-00804-9

**Published:** 2021-03-07

**Authors:** Christina Oetzmann von Sochaczewski, Jan Gödeke, Oliver J. Muensterer

**Affiliations:** 1 Klinik und Poliklinik für Kinderchirurgie, Universitätsmedizin Mainz der Johannes Gutenberg-Universität, Mainz, Germany; 2 Sektion Kinderchirurgie, Klinik und Poliklinik für Allgemein-, Viszeral-, Thorax- und Gefäßchirurgie, Universitätsklinikum Bonn, Venusberg-Campus 1, D-53127 Bonn, Germany; 3 Kinderchirurgische Klinik und Poliklinik, Dr. von Haunersches Kinderspital der Ludwig- Maximilians-Universität München, Munich, Germany

**Keywords:** Health services research, Paediatric surgery, Administrative data, Population‐based, Penile surgery, Inpatients

## Abstract

**Background/purpose:**

Circumcisions are among the most frequent operations in children. Health service data on circumcision in the United States has documented an increase in neonatal circumcisions since 2012. We investigated whether a similar effect could be found in Germany, which does not endorse neonatal circumcision.

**Methods:**

We analysed German routine administrative data for operations conducted on the preputium in order to analyse the frequency, age distribution, and time-trends in hospital-based procedures on a nationwide basis.

**Results:**

There were 9418 [95% confidence interval (CI) 8860–10,029] procedures per year, of which 4977 (95% CI 4676–5337) were circumcisions. Age distributions were highly different between both circumcisions (van der Waerden’s χ² = 58.744, *df* = 4, *P* < 0.0001) and preputium-preserving operations (van der Waerden’s χ² = 58.481, *df* = 4, *P* < 0.0001). Circumcisions were more frequent in the first 5 years of life and above 15 years of age, whereas preputium-preserving procedures were preferred in the age groups between 5 and 14 years of age. The number of circumcisions and preputium-preserving operations decreased in absolute and relative numbers.

**Conclusions:**

The increasing trend towards neonatal circumcision observed in the United States is absent in Germany. The majority of patients were operated after the first year of life and absolute and relative numbers of hospital-based procedures were decreasing. Other factors such as increasing use of steroids for the preferred non-operative treatment of phimosis may play a role. As operations in outpatients and office-based procedures were not covered, additional research is necessary to obtain a detailed picture of circumcision and its surgical alternatives in Germany.

**Level of evidence:**

III.

## Background

Circumcision beyond infancy is among the most frequent paediatric surgical operations in countries that do not encourage neonatal circumcision [1], contrary to those who do [2]. Just recently, the current situation of circumcision in the United States has been described by an increasing proportion of neonates who underwent circumcision. Affirmation of this practice in the guidelines by the American Academy of Paediatrics in 2012 may play a role, as *Many *et al. noted [2]. The guideline of the American Academy of Paediatrics has been criticised, because of a cultural bias that prohibits its applicability in other countries of the developed world [3]. Although this cultural aspect has been recognised before and named the “uniquely American medical enigma” [4]—it is likely that the results of the study by Many et al. [2] will not be transferrable to countries outside the United States. We therefore analysed German routine administrative data for paediatric patients that were treated by circumcision or its preputium-preserving alternatives irrespective of the operating department in order to report data for a country that does not endorse neonatal circumcision.

## Methods

### Routine data from German hospital reimbursement

We bought data files—in the form of separate Microsoft Excel sheets for every included year—from the *Statistisches Bundesamt* (Federal Statistics Office) including principal diagnoses and procedures of the German Modification of the International Classification of Diseases—Version 10 for the years 2005–2017. Data were analysed all operations conducted on the preputium (5–640) with its respective sub-classifications: frenulotomies (5–640.0), dorsal slit (5–640.1), circumcision (5–640.2), frenulum- and preputioplasty (5–640.3), and freeing of preputial adhesions (5–640.5), whereas repositions of a paraphimosis under anaesthesia (5–640.4) were excluded. These data files are stratified in the mandatory age-groups of all administrative statistics: The first year of life, the ages 1–4, 5–9, 10–14, and 15–19 years. The properties and pitfalls of these data have been described in detail elsewhere [5]. In brief, the national hospital statistic became mandatory in 2002 for every hospital that is reimbursed via the German system of diagnosis related groups, any licenced hospital with all its somatic departments, excluding only psychiatric departments, and can be obtained from 2005 onward [6]. Due to the mandatory nature, missing data for discharge information—principal diagnoses, procedures, age, sex, and length of stay—of hospitalised patients are almost non-existent [6]. Coding of these data follows standardised formats. Non-coded or incorrectly coded procedures lead to lack of reimbursement [6].

### Statistical analysis

Statistical analysis was conducted in R (version 3.5.3) [7] with its generic stats4-package (version 3.5.3) unless indicated otherwise. Procedures per year were evaluated using ordinary least square linear regression as described before for these types of data [8–10]. Linear regression’s prerequisites of normality of residuals was checked using the Shapiro–Wilk test and homogeneity of variances was tested via the *F*-test using R’s olsrr-package (version 0.5.3) [11]. Differences between age groups were assessed using van der Waerden’s test [12] followed by posthoc comparison via Conover–Iman test with the PMCMRplus-package (version 1.4.4) [13] as recommended elsewhere [14]. Confidence intervals for point estimates were calculated via bias-corrected, accelerated bootstrap with 10,000 repetitions using the *groupwise Mean*-function from the car-package (version 2.0.0) [15] as described before [16, 17]. Testing for differences between the relative share of circumcisions and its alternatives was done with Student’s *t*-test. Corrections for multiple testing were conducted according to Benjamini–Hochberg [18, 19]. The colour palettes from the viridis-package (version 0.5.3) have been used to generate figures as inclusive as possible for colour-blind readers [20].

## Results

**Fig. 1:**
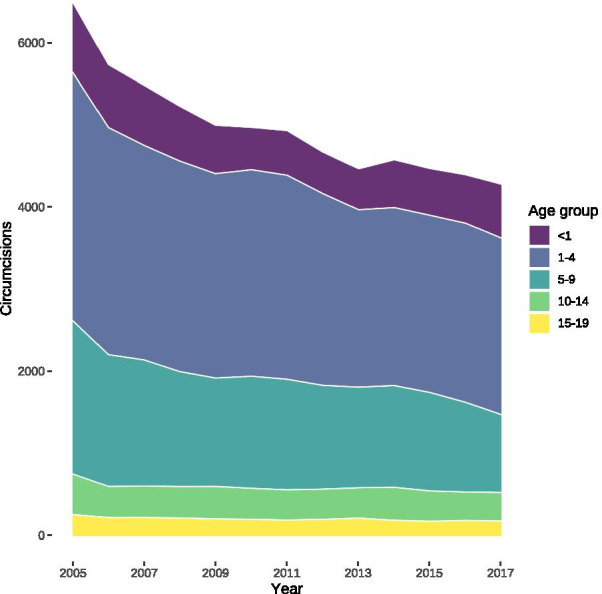
Cumulative number of circumcisions performed per year in Germany in paediatric age groups between 2005 and 2017

**Fig. 2:**
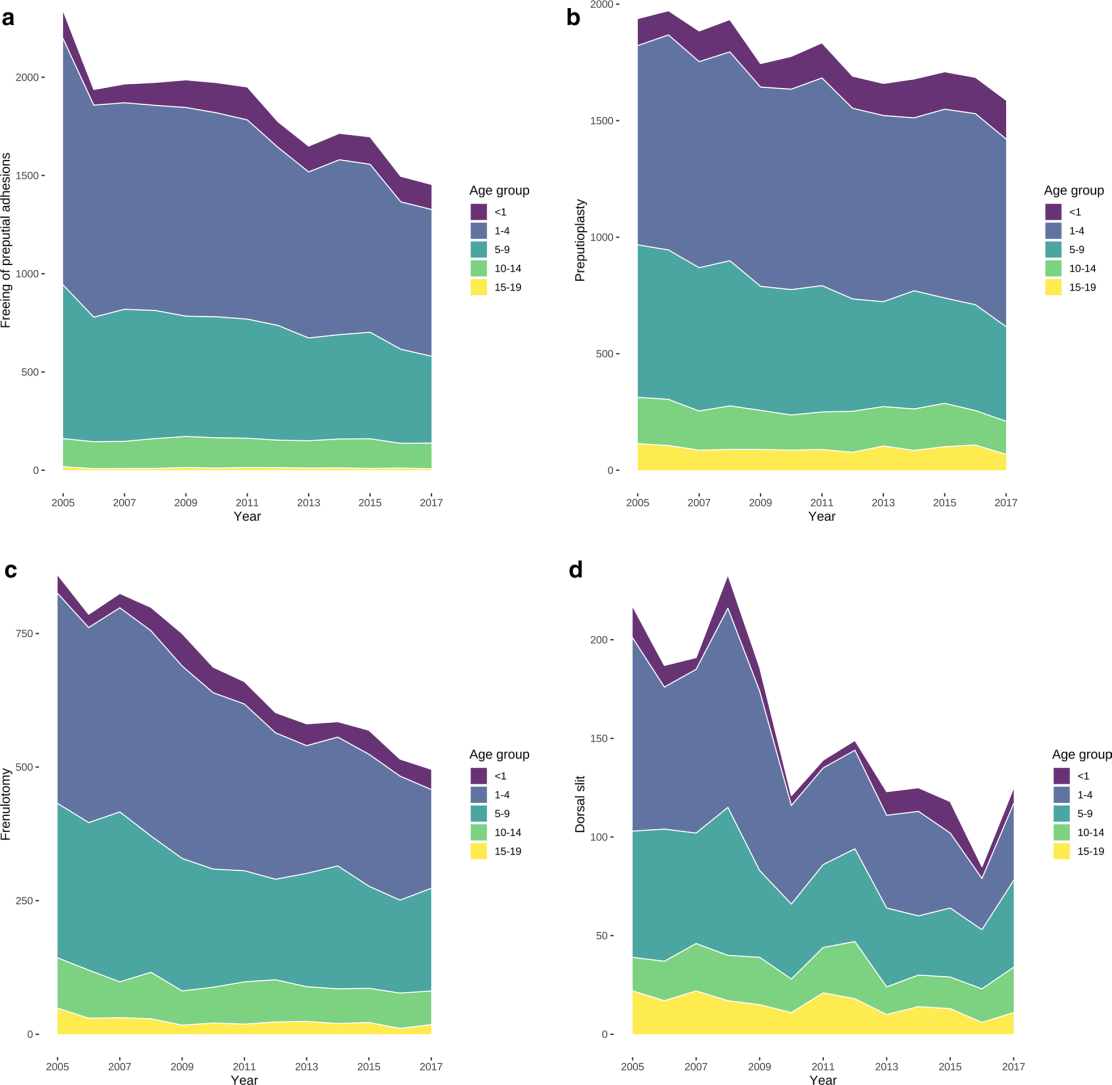
Cumulative number of preputium-preserving alternative procedures per year in paediatric age groups between 2005 and 2017. **a** Freeing of preputial adhesions. **b** Preputioplasty. **c** Frenulotomy. **d** Dorsal slit

There were 122,431 operations on the male prepuce included between 2005 and 2017, which equals 9418 (95% confidence interval: 8860–10,029) procedures per year. The majority of these operations were circumcisions, of which 4977 (95% confidence interval: 4676–5337) were performed annually (Fig. 1). The most frequent preputium-preserving operation was freeing of preputial adhesions with 1840 (95% confidence interval: 1717–1966), followed by 1777 (95% confidence interval: 1713–1844) cases of preputioplasty,  670 (95% confidence interval: 607–734) frenulotomies, and 154 (95% confidence interval: 132–178) dorsal slits (Fig. 2) annually.

We found relevant differences in the distribution of cases among the age groups for both circumcisions (van der Waerden’s χ² = 58.744, *df*  =  4, *P* < 0.0001 with all posthoc comparisons *P* < 0.0001; Table 1) and for the preputium-preserving operations (van der Waerden’s χ² = 58.481, *df * = 4, *P* < 0.0001 with all posthoc comparisons *P* < 0.0001; Table 2).

**Table 1:** Distribution of yearly circumcisions between the age groups

Age group	Mean	95% CI
< 1	621	572–688
1–4	2430	2310–2590
5–9	1330	1230–1480
10–14	383	370–415
15–19	207	197–221

*CI* confidence interval

**Table 2:** Distribution of yearly preputium-preserving operations between the age groups

Age group	Mean	95% CI
< 1	319	291–335
1–4	2170	2030–2320
5–9	1400	1290–1520
10–14	408	392–424
15–19	143	134–159

*CI* confidence interval

The comparison of relative shares of circumcisions and preputium-preserving operations between the age groups revealed that circumcision was more frequent in the first year of life, in the age-groups between 1 and 4 years, and between 15 and 19 years of age (all *P* < 0.0001; Table 3), whereas preputium-preserving operations were more frequent between the older age groups of 5–9 and 10–14 years (both *P* < 0.0001; Table 3).

**Table 3:** Relative share of operations between the age groups

Age group	Circumcision (%)	Preputium-preserving (%)	Difference (%)	95% CI
< 1	65.7	34.3	31.4	26.7–36.2
1–4	52.9	47.1	5.7	4.9–6.7
5–9	48.8	51.2	– 2.4	– 3.5 to – 1.4
10–14	48.4	51.6	– 3.2	– 4.7 to – 1.8
15–19	59.3	40.7	18.5	16.7–20.3

*CI* confidence interval

**Fig. 3:**
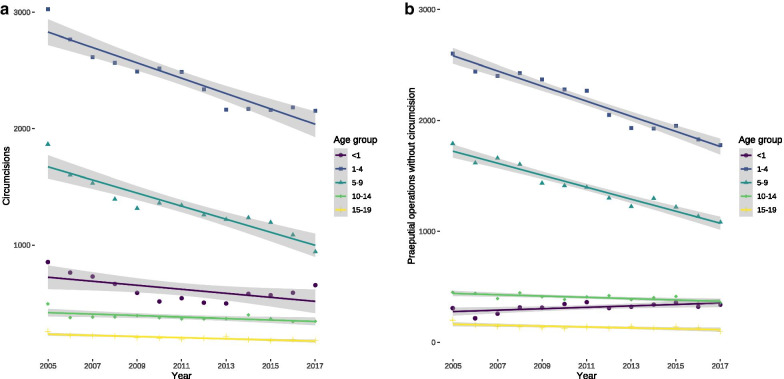
Hospital-based circumcisions and preputium-preserving operations between 2005 and 2017. **a** Circumcisions. **b** Preputium-preserving operations. Their numbers decreased between 1 and 4 years of age by 68 per year (95% CI 58–78, *P* < 0.0001), between 5 and 9 years by 54 yearly procedures (95% CI 46–63, *P* < 0.0001), from 10 to 14 years of age by 6 operations per year (95% CI 2–9, *P* = 0.0035), and between 15 and 19 years by 4 procedures (95% CI 1–7, *P* = 0.0075), but increased in the first year of life by 6 yearly procedures (95% CI 1–12, *P* = 0.0225)

**Fig. 4:**
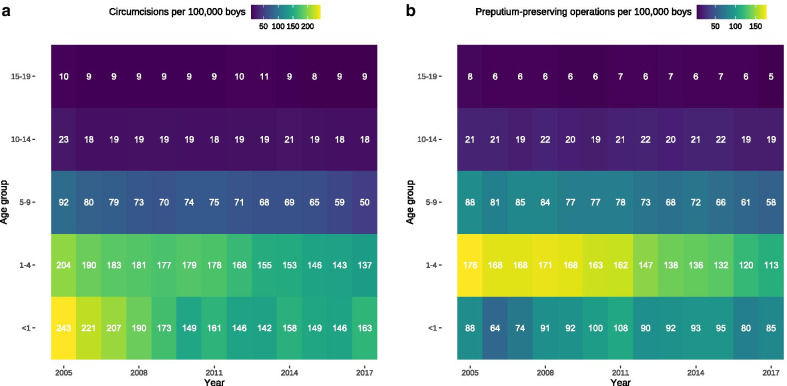
Hospital-based circumcisions and preputium-preserving operations per 100,000 boys between 2005 and 2017. **a** Circumcisions. **b** Preputium-preserving operations

There was a relevant decrease of circumcisions among patients in all age groups between 2005 and 2017 (Fig. 3a), which was also true for preputium-preserving operations with the exception of the first year of life whose numbers increased over time (Fig. 3b). This increase in absolute numbers of procedures in the first year of life could be related to an increasing number of live births since 2011, because the numbers of circumcisions per 100,000 boys in their first year of life decreased (Fig. 4a) and remained stable for preputium-preserving operations (Fig. 4b). Similar to absolute numbers, the relative numbers of procedures also dropped in the remaining age-groups for both circumcision (Fig. 4a) and its preputium-preserving alternatives (Fig. 4b).

## Discussion

A recent study demonstrated an increasing frequency of neonatal circumcisions in the United States following publication of the most recent American Academy of Paediatrics guideline on the subject from 2012, which expresses an affirmative stance on neonatal circumcision after more than a decade of equipoise [2]. Interestingly, a similar trend has been described even before the millennium using routine hospital data [21]. In preceding studies, two moderator variables have been identified: Medicaid coverage of circumcision was linked to a 24% higher rate of neonatal circumcision compared to non-Medicaid-coverage, and Hispanic descent was associated to lower circumcision rates [22]. Consequently, the number of neonatal circumcisions dropped in states that withdrew Medicaid coverage for neonatal circumcisions, but remained stable in states that did not [23]. Likewise, this study also demonstrated a reduced frequency of neonatal circumcision among children of Hispanic descent and a slightly reduced frequency of neonatal circumcisions among non-Hispanic black children [23]. For Germany, circumcision has been associated to being born into a migrant family, particularly being descended from Turkish families with a more than tripled rate of circumcisions compared to the rest of Germany’s paediatric population [24]. Many et al. [2] used the Paediatric Health Information System database that covers free-standing children’s hospitals from many states from the United States of America, although particularly those in metropolitan areas, and has been described to be somewhat representative for the whole paediatric population in the United States of America [25]. Of note, the moderator variables were associated with a reduced frequency of neonatal circumcisions, but not an increase, which has not been described for any other potential factor [22, 23].

This is in contrast to other countries such as Australia for which a decreasing rate of circumcision has been described in the same time period [26] and beyond [27]. Similar trends were observed in England [28] and Northern Ireland [29], but large-scale population-based data is scarce and reasons remain unclear. Using nationwide administrative data for hospital reimbursement, we were also able to show a decreasing trend in circumcisions in German hospital-based procedures among all age groups. However, the preputium-preserving operations also showed a declining trend in all age groups, except those in their first year of life. This could be linked to the general reduction in surgical diseases described before, for example in infantile hypertrophic pyloric stenosis [30] or inguinal hernia [31]. An alternative explanation might be a shift of these operations towards outpatient surgery: The vast majority of paediatric surgical departments offer circumcision as an outpatient procedure [32], but this is unlikely as these procedures have been conducted primarily on outpatients even back in the 1980s [33] as it was the case in the United States, too [34]. Although there is no universally accepted age threshold that would preclude outpatient surgery, in Germany, it is uncommon below the age of six months. An individual decision between caretakers, surgeons, and anaesthetists should be reached on whether the procedure can be performed as an outpatient surgery [35]. Consequently, we are confident that the present data covers all operations in neonates, except ritual circumcisions performed by non-physicians, but there is considerable uncertainness in older age groups. This is supported by a cohort of boys that received a preputial operation from an office-based paediatric surgeon, in which none of the patients were operated on in their first year of life [36].

The obvious difference of our data to those by Many et al. is the age distribution between the two cohorts: They reported 67% of all circumcisions in the first year of life, whereas only 12.5% of circumcisions in our cohort were performed in this age group. For the preputium-preserving operations, this number was even smaller with 7.2% of all procedures. This difference is not surprising as the German guideline only recommends circumcision for Lichen sclerosus and high-grade urologic malformations in order to prevent recurrent infections, which leaves only phimosis as a possible indication for surgery and only after a local therapy with steroids has been applied for a sufficient amount of time [37]. Similarly to the observation by Many et al. [2] for circumcisions, the effects of guideline changes could also be observed in Germany, but the other way round: The first step of a local treatment with steroids [37] resulted in a directly observable change in therapy preferences. In 2005, local steroids were never used, but became the definitive treatment for the majority of patients in a single-centre analysis from a tertiary paediatric surgery unit [38]. A result that has been validated in later cohorts [39] and thus reflects the different recommendations made by the German guidelines compared to the guideline by the American Academy of Paediatrics: An affirmative stance towards neonatal circumcision [40] versus primary non-operative treatment that considers a primary circumcision without preceding local steroid therapy in the absence of a balanitis xerotica obliterans or high-grade urinary tract anomalies as not *lege artis *[41]. For the United States of America, there is no recommendation by the American Urological Association towards the use of topical steroids for phimosis [42]. However, results from a cohort treated by an office-based paediatric surgeon before 2015 showed that only 11% of operated boys received preceding treatment with local steroids [36], so guideline compliance may vary between hospital- and office-based surgeons. On the contrary, in a cohort from the Capital region of Denmark, the majority of patients received topical steroids as first-choice treatment of phimosis and had a similar age-distribution as in the our report using administrative data, too [43].

Although ritual circumcisions should in theory not be conducted within the health-care system, it is likely that they are present [44]. Their relevance has been quantified in data from England based on the comparison with the expected incidence of phimosis [28]. Based on the report that in a German cohort of 176 boys treated for phimosis by an office-based paediatric surgeon, the decision for treatment was based on a non-retractable preputium [36], and taking into account that a non-retractable foreskin is present in 50% of first-graders [45], it is unclear how many circumcisions were performed for therapeutic reasons. Non-medically indicated circumcision in boys in Germany can be performed explicitly by discretion of parental custody since 2013. In the first six months of life, circumcision may be performed by non-physicians if this person is designated to do so by a religious community [46]. Consequently, the latter cases will not be represented within our data if there is no immediate major complication that required therapy on an inpatient basis. Currently, there is no estimate on the number of these procedures conducted by non-physicians without adequate anaesthesia [47]. This might be a general issue due to, perceived, profanity of topics like circumcisions, exemplified by unpublished theses with relatively large cohorts examining the efficacy of local steroids [38, 39], whereas small cases series with single digit numbers of patients still easily appear in the international literature if they present something novel or deal with more prestigious diseases [48]. This is important in so far, as truly representative cohort studies found a more than doubled prevalence of circumcision in boys from families with a migrant background and even a more than tripled prevalence in those from Turkish families [24]. This suggests that ritual circumcisions frequently occur.

A major limitation of our data is its sole focus on hospital-based procedures. As described before, circumcision and its alternatives are often conducted on outpatients [33, 34] and office procedures are recompensed to the surgeon via a different way than it is the case for hospitals. Consequently, our data does not offer the full picture of circumcision and its preputium-preserving alternatives as the distribution of procedures may be different in outpatients. The generalisability to other countries that do not endorse neonatal circumcision is therefore unclear due to the different structure of the German health system that favours inpatient-based approaches due to highly different reimbursements compared to office-based procedures [49]. Another limitation is the focus of administrative data used by us on cases instead of patients: The same patient treated with a preputium-preserving procedure might later on reappear in the data with a circumcision, but this cannot be tracked due to the case-based approach of the data. It may thus also be possible that there is no reduction in foreskin surgery, but just a shift towards outpatient-clinics or office-based surgeons, because these procedures cannot be tracked by the administrative data used by us. This limitation would not apply to databases of the statutory health insurances, because they are patient-based instead of case-based. Other limitations are those that are inherent in the use of secondary administrative datasets: Lack of clinical and demographic details due to the focus on reimbursement of hospitals, misclassifications due to coding errors, and systematic errors introduced by variations in coding practice, because the coding is done at the local hospital level.

Other factors such as information of parents on the altered risk of sexually transmitted diseases following circumcision are unlikely to have effects on parental decisions for or against circumcision [50]. Likewise, we also consider potential effects of a vaccination against human papilloma virus diminishable, because this vaccination is only among the lists of services covered from 2019 onwards by the statutory health insurances in Germany that serve around 90% of Germany’s population [51].

However, our study offers a first comparator to the study by Many et al. [2] from a country that does not endorse neonatal circumcision despite its limitations. Moreover, it might serve as a vanguard to prompt others to contribute population-based data on circumcision and its preputium-preserving alternatives as it has been done for infantile hypertrophic pyloric stenosis [30] or inguinal hernia [52].

## Conclusions

An increasing trend towards neonatal circumcision observed in the United States is absent in Germany. The vast majority of patients were operated after the first year of life for both circumcision and preputium-preserving procedures. The number of both types of procedures was declining over time with the exception of preputium-preserving procedures in the first year of life. Topical steroid therapy as recommended in the local guidelines may play a role in this development. Additional population-based data from countries able to cover both in- and outpatients in their respective registries or administrative data are needed to gain further insight into the epidemiology of these operations. For data from Germany, a combination of hospital-based and office-based procedures is necessary to gain a full insight of the spectrum of preputial surgery. These data might be available at the databases of the statutory health insurances, at least for outpatients operated in hospitals, which would also offer the opportunity to explore the diagnoses that represent the reasons for the surgical procedures.

## Data Availability

The data supporting the findings of this study are publicly available if licenced with costs from the *Statistisches Bundesamt* (Federal Statistics Office) [53].
